# Narrowing the health equity gap. How can implementation science proactively facilitate the cultural adaptation of public health innovations?

**DOI:** 10.1007/s00103-025-04057-x

**Published:** 2025-05-21

**Authors:** Zoe Fehlberg, Marlena Klaic, Samantha Croy, Stephanie Best

**Affiliations:** 1 Melbourne School of Health Sciences, The University of Melbourne, Melbourne, Australia; 2 Centre for Population Genomics, Murdoch Children’s Research Centre, Melbourne, Australia; 3 Centre for Population Genomics, Garvan Institute of Medical Research, University of New South Wales, Sydney, Australia

**Keywords:** Innovation, Unintended consequences, Implementation science, Health equity, Cultural adaptation, Innovation, Unbeabsichtigte Folgen, Implementierungswissenschaft, Gesundheitliche Chancengleichheit, Kulturelle Anpassung

## Abstract

While the ambitions of innovation in public health are usually geared towards improving health outcomes, an unintended consequence of the innovation process is that it can exacerbate health inequity. People who are disproportionately excluded from accessing the benefits from innovations in public health include, among others, minoritised racial and ethnic communities. Advancing racial and ethnic health equity by centring attention on systemic factors influencing health inequalities—for example, how structural racism influences public health—has gained much focus among researchers, including those in implementation science. Implementation science is a field ideally placed to actively intervene and enhance an equitable process of innovation. One of the key pieces in working towards implementing public health innovation that promotes health equity is progressing the science of adaptation. Cultural adaptation requires systematic changes to an intervention, context, or implementation activity to ensure the relevance and fit for a population, whilst retaining fidelity to the evidence-based components. In this discussion, we propose five implementation science approaches to proactively facilitate cultural adaptation in public health innovation and promote health equity. We discuss (1) structuring cultural adaptation through a formal process and (2) using theory, (3) incorporating inclusive and participatory approaches to cultural adaptation, (4) conceptualising cultural adaptation as an implementation strategy, and (5) investigating cultural adaptation to implementation science data collection tools. Further, we use an example of a precision public health program to exemplify a framework for reporting and making adaptations. Implementation science can use the practice of adaptation as part of the inclusive and equity-driven approaches to the implementation of public health innovation.

## Abstract (de)

Während die Innovationsbestrebungen im Gesundheitswesen in der Regel auf eine Verbesserung der Versorgung abzielen, besteht eine unbeabsichtigte Folge des Innovationsprozesses darin, dass er gesundheitliche Ungleichheiten verschärfen kann. Zu den Menschen, die unverhältnismäßig stark von den Vorteilen der Innovationen im Gesundheitswesen ausgeschlossen sind, zählen unter anderem Angehörige von ethnischen Minderheiten. Die Förderung der gesundheitlichen Chancengleichheit von Menschen unterschiedlicher Ethnien durch die Konzentration auf systemische Faktoren, die gesundheitliche Ungleichheiten beeinflussen, z. B. wie struktureller Rassismus die öffentliche Gesundheit beeinflusst, hat bei Forschern, auch in der Implementierungswissenschaft, viel Aufmerksamkeit erlangt. Die Implementierungswissenschaft ist ein Bereich, der ideal geeignet ist, um aktiv einzugreifen und einen gerechten Innovationsprozess zu fördern. Einer der wichtigsten Aspekte bei der Umsetzung von Innovationen im Bereich der öffentlichen Gesundheit, die die gesundheitliche Chancengleichheit fördern, ist die Weiterentwicklung der Forschung im Bereich der Anpassung. Kulturelle Anpassung erfordert systematische Änderungen an einer Intervention, einem Kontext oder einer Umsetzungsaktivität, um die Relevanz und Eignung für eine Bevölkerung sicherzustellen, während die evidenzbasierten Komponenten beibehalten werden. In dieser Diskussion schlagen wir fünf Ansätze der Implementierungswissenschaft vor, um die kulturelle Anpassung bei Innovationen im Bereich der öffentlichen Gesundheit proaktiv zu erleichtern und die gesundheitliche Chancengleichheit zu fördern. Wir erörtern (1) die Strukturierung der kulturellen Anpassung mithilfe formeller Prozesse und (2) die Verwendung von Theorien, (3) Einbeziehung inklusiver und partizipativer Ansätze zur kulturellen Anpassung, (4) Konzeptualisierung der kulturellen Anpassung als Implementierungsstrategie und (5) Untersuchung der kulturellen Anpassung an die Datenerhebungsinstrumente der Implementierungswissenschaft. Darüber hinaus verwenden wir ein Fallbeispiel zur Präzisionsgesundheit im Bereich Public Health, um einen Rahmen für die Berichterstattung und Anpassungen zu veranschaulichen. Die Implementierungswissenschaft kann die Praxis der Anpassung als Teil inklusiver und auf Chancengleichheit ausgerichteter Ansätze zur Umsetzung von Innovationen im Bereich der öffentlichen Gesundheit nutzen.

Innovation is critical for advancing medical and health research with the aim of solving a problem and driving health gains. Innovation is a process and, in the context of health care, can be found in multiple formats, ranging from digital health interventions to drug development and new service design [1]. While the ambitions of innovation in health care are usually aimed at improving care, the inverse-care law highlights an unintended consequence of the innovation process: Health benefits from innovation are rarely distributed equitably across population groups [2]. Simply, innovation can reinforce pockets of excellence that continue to serve more advantaged groups. Groups who are disproportionately impacted include, among others, minoritised racial and ethnic communities, Indigenous peoples, socioeconomically disadvantaged groups, and people with disabilities [3].

Diffusion of innovation theory attests to the inverse-care law phenomenon and theorises that the spread of innovation begins with uptake by a small group of ‘early adopters’ who are passively influenced by the characteristics of the innovation, communication channels, time, and a social system [4]. When the influence of a social system on the diffusion of innovation among racial and ethnic communities is being considered, the interrelated impacts of colonisation and racism cannot be ignored. For example, critical race theory conceptualises that racialised disparities, including those related to health, are not born of individual ill-intention but rather by the historical and contemporary interpersonal, social, and political processes, structures, and systems operating as intended to maintain the status quo [5]. The impacts of structural racism are socially and financially costly because they deplete trust, create barriers to health services, and lead to poorer health outcomes [6, 7].

As characterised by the Framework for Public Health Innovation, dissatisfaction with the status quo frequently initiates a need for public health innovation [8]. However, public health innovation is not immune to unintended consequences. The COVID-19 pandemic exemplified racialised health disparities and, importantly, showed the gains made when innovative approaches that promoted equity and addressed structural racism were used [9]. For example, uptake of vaccines was positively impacted when multidisciplinary and community-based collaborations were used to inform and co-design adaptations to vaccine rollout programs, including having drop-in information sessions attended by community champions (e.g. business leaders and people from faith groups and social clubs), having an appropriate workforce (e.g. bicultural workers at vaccine centres), and translating information material into different languages [10, 11]. The pandemic showed that by putting in place strategies that respond to the strengths of the community, better health outcomes can be achieved, and these principles and practices can be applied more widely to tackle unintended consequences.

In contrast to passive diffusion of innovation, actively intervening in the innovation process is imperative for implementing equitable health care innovation among diverse populations and minimising the risk of unintended consequences. Implementation science is a field established with a vision to use theory and systematic methods to ‘facilitate widespread and equitable adoption, delivery and sustainment of scientific advancements’ [12]. The vision underscores the field’s role within adoption and diffusion of evidence-based innovation and the ambition to uphold principles of health equity. Health equity is the commitment to reduce and eliminate unfair and avoidable differences in a person’s health and access to health resources among groups [13]. As reflected in the exponential increase in the number of equity-focused publications, health equity is becoming a core tenet and subject of research and discussion in implementation science [14, 15]. With a shared ambition to ensure that benefits are fairly distributed, the coalescence of implementation science and public health has grown [16–19]. Commonalities aside, more can be done to establish how the two can work to narrow, rather than widen, the equity gap within public health innovation.

Recognising that innovation is implemented into systems in which health inequities relating to race and ethnicity exist, calls have been made for no implementation without cultural adaptation [20]. In this discussion, we outline five approaches, and provide a case example, to show how implementation science can proactively facilitate cultural adaptation in public health innovation and promote health equity.

## Adaptation as an opportunity for implementation science to intervene in the innovation process to promote health equity

One key piece, among others highlighted in the literature, in working towards implementing public health innovation that promotes health equity is progressing the science of adaptation [21]. In implementation science, the term ‘adaptation’ refers to the systematic process of prospectively, concurrently, and retrospectively altering aspects of either the intervention content, context, evaluation process, or implementation activity to improve the relevance and fit, whilst retaining fidelity to the evidence-based components [22]. Cultural adaptation requires specific consideration around language, culture, and context to improve the compatibility of an evidence-based intervention with a group’s cultural values, norms, customs, beliefs systems, and ways of making meaning [23]. Cultural adaptation to an intervention can range from ‘surface’-level changes, e.g. translation of materials or images to improve how the intervention is perceived, to ‘deep structural’ changes, e.g. understanding and incorporating cultural and contextual experiences of the relevant communities into protocol development [24].

**Fig. 1:**
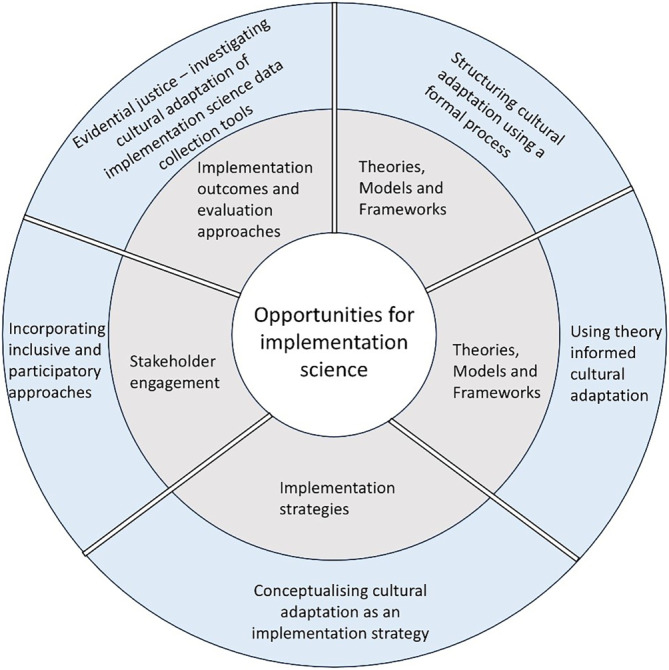
Five implementation science approaches to proactively facilitate cultural adaptation in public health innovation and promote health equity. Each approach (outer ring) relates to a central component of implementation science (inner ring) [26]. (author’s own figure)

Given that an evidence-based intervention rarely fits perfectly when taken into new contexts or populations [4], adaptation is a necessary component of implementation and, thus, a focus of implementation science [25]. Adaptation is also a focus of public health, as public health efforts are often focused on replicating interventions in new settings or populations. This discussion centres five implementation science approaches to facilitate cultural adaptation in public health innovation (Fig. 1). Each approach (the outer ring) relates to a core element of implementation science identified for addressing structural racism (the inner ring) [26].

### Structuring cultural adaptation through a formal process

One of the opportunities for implementation science to intervene in the innovation process is structuring cultural adaptation through a formal process or method (e.g. literature review, robust and meaningful participation from stakeholder groups that include the potential beneficiaries). Doing so has the potential to produce more impactful outcomes and generate transferable evidence [25]. Numerous ‘tools’ exist across various research fields that can be used to guide cultural adaptation [27–29]. As summarised in Table 1, these tools have different purposes and include, but are not limited to, tools that identify factors that indicate when to adapt and the reason for cultural adaptation (e.g. Lau’s model [30]), tools that outline which intervention components are adaptable (e.g. the ecological validity model [31]), tools that guide the process of adaptation (e.g. the cultural adaptation checklist [32]), and tools that promote documentation of the adaptation process either retrospectively (e.g. FRAME, the framework for reporting adaptations and modifications–enhanced [22]) or prospectively while measuring impact (e.g. model for adaptation design and impact [33]). Although not explicitly focused on cultural adaptation, the model for adaptation design and impact [33] spans three domains to help users report adaptations and, importantly, think through possible intended and unintended impacts on important outcomes. Domain 1 details several adaptation characteristics (e.g. constructs related to ‘what, who, and when’ to promote consistent reporting of adaptation) and comparisons of findings across settings. Domain 2 outlines possible mediating (i.e. those that align to the core function of the intervention) or moderating (e.g. ‘why, how, and under what’ circumstances positive impact would be more likely) factors. Domain 3 evaluates the impact of the adaptation on established implementation outcomes (e.g. acceptability, feasibility, adoption, cost, appropriateness, penetration, fidelity, and sustainability) and service or client outcomes. Each of the tools has value, and some have consolidated aspects; for example, the ecological validity model is incorporated into other tools (e.g. the ecological validity model is incorporated into tools, such as Perera’s 4 Step Process [20] or The Cultural Adaptation Checklist [32]).

**Table 1:** Examples of types of cultural adaptation tools and their value-add for implementation science

Purpose	Example of tool	Summary of tool	Value-add for implementation science
When: *Determine when to adapt and reason for it*	Lau’s model [30]	**Adaptation required when evidence suggests that** (1) A new population has distinctive and unique sociocultural context or risk and resilience that require consideration or addition of new treatment elements to address contextual issues and improve fit (2) The social validity of an intervention is compromised, reducing engagement, exposure, and effect	Permits investigation for previously excluded communities
		**Approaches to adaptation** (1) Contextualising intervention content (2) Enhancing engagement	
What: *Outline adaptable intervention dimensions*	Ecological validity model [31]	**Eight dimensions** Language, persons, metaphors, content, concepts, goals, methods, and context	Supports wide-reaching investigation across contextual settings
How: *Outline the adaptation process*	Integrated strategy for cultural adaptation of interventions [37]	**Three-step process** (1) Assess community beliefs: *Information gathering through community engagement about the community’s experience of the health problem and relevant determinants* (2) Examine the interventions’ fit with the community’s beliefs: *Engage community to select acceptable evidence-based interventions that address factors identified in step 1* (3) Adapt interventions: *Gather information on resources required for implementation, as well as adaptable aspects of the evidence-based intervention*	Offers formal steps to support selection and in-depth analysis
How: *Outline the adaptation and evaluation process*	Perera’s four-step process [20]	**Four-step process** (1) Information gathering: *Conduct a rapid desk review to gather relevant preexisting information (e.g. demographic, socioeconomic, help-seeking patterns, coping mechanisms)* (2) Adaptation hypotheses: *Revise existing intervention protocols to identify components for adaptation based on the ecological validity model* (3) Local consultations: *Develop a focus group discussion guide based on step 2 and use it to interview local specialists, community members, and implementers to elaborate and/or validate previous findings* (4) External evaluation: *Engage reviewers in the evaluation of the intervention protocol using the cultural relevance questionnaire*	Progresses from extant literature to in-person consultation
How: *Outline the adaptation and implementation process*	Cultural adaptation checklist [32]	**Checklist to guide and appraise the quality of adaptation based on the ecological validity model dimensions** (1) Language: Four questions to assess the planning and implementation process (2) Persons: Four questions to assess the planning and implementation process (3) Content: Five questions to assess the planning and implementation process (4) Goals: Four questions to assess the planning and implementation process (5) Methods: Six questions to assess the planning and implementation process (6) Context: Four questions to assess the planning and implementation process (7) Process: Five questions to assess the planning and implementation process	Poses questions to assist with planning and implementation
How: *Outline the adaptation and implementation process*	Mental health cultural adaptation and contextualization for implementation (mhCACI) procedure [38]	**Ten-step process** (1) Identify mechanisms of action (2) Conduct a literature desk review for the culture and context (3) Conduct a training-of-trainers (4) Translate intervention materials (5) Conduct an expert read-through of the materials (6) Perform qualitative assessment of intervention population and site (7) Conduct practice rounds (8) Conduct an adaptation workshop with experts and implementers (9) Pilot-test the training, supervision, and implementation (10) Review through process evaluation	Offers guidance to improve scalability to other contexts
Describe: *Retrospectively document the adaptation process*	Framework for reporting adaptations and modifications–enhanced (FRAME) [22]	**Eight questions to guide retrospective reporting** (1) When and how in the implementation process the modification was made (2) Whether the modification was planned/proactive (i.e. an adaptation) or unplanned/reactive (3) Who determined that the modification should be made (4) What was modified (5) At what level of delivery the modification was made (6) Type or nature of context or content-level modifications (7) Extent to which the modification is fidelity-consistent (8) Reasons for the modification, including (a) the intent or goal of the modification (e.g. cultural adaptations, to reduce costs, etc.) and (b) contextual factors that influenced the decision	Provides a practical tool for documenting adaptation broadly, including cultural factors
Describe: *Prospectively document the adaptation process and impact*	Model for adaptation design and impact (MADI) [33]	**Three domains to guide prospective reporting and considerations for the potential impacts** (1) Adaptation characteristics (based on the FRAME domains), e.g. what, nature, who, for whom, when (2) Possible mediating (fidelity consistent) or moderating factors (goal/reason, systematic, proactive) (3) Implementation and intervention outcomes. Evaluate the impacts (intended and unintended) on established implementation, service, and client outcomes	Encourages anticipation around unintended and intended impacts
Theory: *Incorporate a broader structural approach to the implementation of innovation*	Antiracism lens to core elements of implementation science [26]	**An antiracism lens applied to five core elements of implementation science** (1) Stakeholder engagement: *Necessitates early and ongoing inclusion and engagement of all communities and stakeholders affected by research outcomes as foundational* (2) Conceptual frameworks and models: *Consider racism as a determinant and key aspect of context in implementation science conceptual frameworks, theories, and models* (3) Development, selection, and/or adaptation of evidence-based interventions: *Consider the development, selection, and/or testing of multilevel and structural interventions that include a focus on promoting health equity and addressing racism, as well as the de-implementation and dismantling of harmful or inequitable policies, practices, programs* (4) Evaluation approaches: *Explicitly include measures and evaluation approaches to assess racism and health equity* (5) Implementation strategies: *Apply and test implementation strategies to advance the spread and scale of antiracist, equity-focused solutions*	Supports holistic thinking around the adaptation process

Despite their availability, cultural adaptation tools are not widely or sufficiently used, leaving the description and documentation of cultural adaptation poorly understood and limiting generalisable learnings across settings [34]. For example, in a review of 41 studies reporting the implementation of a diabetes prevention program through lifestyle intervention, Tabak et al. describe the various translational strategies and cultural adaptation made to improve the implementation of the program [35]. The authors found that when mapped to the eight dimensions of the ecological validity model (language, persons, metaphors, content, concepts, goals, methods, and context), the most common cultural adaptation was made to the content of intervention materials, such as providing culturally relevant recipes and exercise activities. Including bilingual or community members as part of the program (persons) was another frequently used dimension of cultural adaptation. The authors also reported limited evidence that the adaptations were evaluated against implemented outcomes beyond adoption or feasibility [35]. The implication of these results shows room for improvement to include other important aspects of cultural adaptation and indicates how structuring adaptation using a framework such as the ecological validity model—or, indeed, tools that incorporate different purposes—could assist by prompting consideration of a wider range of theoretically relevant concepts and evaluation against a wider range of important outcomes, including health equity and racism.

### Using theory-informed cultural adaptation

As mentioned, theory, such as critical race theory or diffusion of innovation theory, can also play a role by providing an understanding of influencing factors on the innovation process, e.g. structural racism or social systems [36]. Beyond providing context to understand how these factors manifest and operate, theory can also be used to guide and evaluate the process of cultural adaptation. For example, Naderbagi and colleagues describe, in an evidence synthesis of the literature on the cultural adaptation of digital health interventions, how having an underlying research ethos used to orient researchers (e.g. cultural humility) forms the centre of a nested and holistic approach to cultural adaptation. The authors describe how using the theory of cultural humility enabled the research team as they co-produced a digital intervention with community to practice continual reflection and open-mindedness to adapt and identify local cultural values different from their own and form strong community relationships. Shelton et al. describe how applying an antiracism lens, which encompasses deliberative efforts to counter systemic racism, to five core elements of implementation science (Table 1) may enable researchers to critically think about and move towards addressing racism within implementation efforts [26].

Given the idea that there should be no implementation without cultural adaptation, we propose that an important research direction will be to determine an approach to help select which of the existing cultural adaptation tools and theories to use. Further, how the tools can be optimised and embed facilitated collaboration with multiperspective stakeholders to promote widespread use within the implementation of public health innovation requires investigation.

### Incorporating inclusive and participatory approaches to cultural adaptation

Importantly, two of the previously mentioned cultural adaptation tools shown in Table 1 include stakeholder engagement [20, 26, 37]. To create impactful adaptation, the process needs to start with inclusive practices that support an inclusive approach to adaptation. Inclusive practices, such as community-based participatory approaches, support stakeholder collaboration with end users, community groups, and other stakeholders (e.g. policymakers, providers, and program developers) with accountability to address power imbalances and facilitate engagement in decision-making [39]. Timing is also important, requiring genuine partnership and engagement of various stakeholders along the life course of implementation research [40]. As proposed in the literature, asking at the outset whose voice is and is not included and how power and resources can be shared are practical first questions [26, 40]. Understanding what would encourage different stakeholders to participate in the process and decision-making would likely vary between groups and may lie in future work to understand what each group would consider valuable. If done well, and if the right people have a seat at the table, it may be likely that adaptation tools could be more effectively utilised to explore the scope of the problem, understand the context and needs of the population, establish ongoing partnerships across the life cycle of the innovation process, and create data for evidence-based decisions. As with embedding participatory approaches throughout the life course of an adaptation to a public health innovation, using co-productive approaches to set smart and measurable goals for health equity within the adaptation process needs to begin during the planning phase and remain throughout resulting stages. This is where implementation science as a field needs to be equipped for genuine and impactful participatory and co-productive approaches to develop/adapt interventions and implementation strategies.

### Conceptualising cultural adaptation as an implementation strategy

Implementation strategies are used to enhance the uptake of evidence-based practices by influencing the interplay between the intervention, the processes of implementation, and the context [41]. Given that implementation strategies and cultural adaptation share mechanistic ambitions, cultural adaptation could be conceptualised as an implementation strategy [21]. The methods used to select and design implementation strategies can vary. However, they are commonly selected using systematic processes to first understand the problem, are designed with stakeholders, and are evaluated against outcomes relevant to implementation, e.g. acceptability, feasibility, and cost [42]. Therefore, framing cultural adaptation as an implementation strategy may be useful to ensure that contextual factors and relevant stakeholders’ voices and needs are incorporated and tested to understand the impact on important outcomes such as acceptability and equity. Lesser known, and an area for further investigation, would be to identify examples of where cultural adaptation has been used as an implementation strategy to support the implementation of evidence-based public health interventions and determine the impact on health and implementation outcomes.

### Evidential justice—investigating cultural adaptation of implementation science data collection tools

Producing meaningful evidence to guide the implementation of innovation may require critically examining what and how evidence is generated and, where necessary, making adaptations to the data collection tool and methods.

In implementation science, there is a strong push to move away from the traditional research-evidence hierarchies in which randomised controlled trials sit at the top. Rather, researchers are encouraged to adopt study designs that are more effective at capturing contextual factors including structural racism, e.g. pragmatic trial designs in which interventions are tested in real-world settings [12]. In addition, participatory approaches and co-design are used in implementation science to ensure that community voices and values are reflected in research [43]. However, a disconnect may remain if the tools used to collect and synthesise evidence when working alongside communities, or as part of controlled study experiments, are derived from disciplinary concepts and frameworks that are culturally misaligned. The consequence is that the disconnect may contribute to evidential injustice, i.e. when the assessment of evidence used in decision-making is unfair, biased, or embodies disciplinary assumptions and forms part of epistemic injustice [44, 45]. Therefore, to be better equipped to capture evidence that is meaningful for all, we consider there to be a real need, beyond rethinking study designs,and using participatory approaches, to also recognise, revise, and reconfigure the data collection tools used to capture evidence in the development and implementation of public health programs.

As outlined in the model for adaptation design and impact, understanding the impact of an intervention on established implementation outcomes—e.g. acceptability, feasibility, adoption, appropriateness, implementation cost, penetration, and sustainability—and interrelated service and client outcomes is central to understanding the intended and unintended consequences of implementation [46]. For example, if community acceptability of an innovation is low, this finding signifies that an unintended consequence may be poor uptake and result in inequity of access. Implementation outcomes may also provide evidential insight into whether an evidence-based intervention remains ‘implementable’, e.g. acceptable, feasible, and sustainable, when scaled out to new contexts and populations or, indeed, whether adaptation is required [47]. Catalogued in an online repository are data collection tools and measures available to assess implementation outcomes (https://implementationoutcomerepository.org). Similarly to cultural adaptation tools, structuring assessment around existing tools is important to produce generalisable evidence [48].

The three outcomes of acceptability, appropriateness, and feasibility are used to understand how individuals think and feel about an intervention [47]. Therefore, to reduce the risk of epistemic and evidential injustice, research is needed to identify whether the data collection tools used to collect and synthesise evidence for the three outcomes are culturally relevant for use with diverse communities. Recognising how the data collection tools have been developed, used, or culturally adapted, and with whom this process has taken place, will be an important first step to investigating whether the outcomes and associated data collection tools are fit for purpose. Doing this will require partnering with diverse communities using participatory approaches and, where necessary, co-producing adaptations to how evidence is generated, or bringing to light other outcomes that are important.

## Putting it all together in practice—case example

Below we detail an example of an implementation science program situated within a public health innovation (OurDNA). The example brings together a framework for reporting adaptations to the OurDNA program and an antiracism approach to generating culturally inclusive outcome data.

### Precision public health: definition

Rapid advances in genomic technologies are driving a genomic approach to precision public health. Precision public health seeks to improve population health by incorporating aspects of precision medicine, such as human genomics, with public health concepts such as prevention and health promotion [49]. Genomic screening programs, such as genomic newborn screening and screening for variants associated with increased cancer risk, are examples of precision public health initiatives [50, 51].

### Equity issue/agenda

Despite great promise, without intentional focus on health equity there is the risk that precision public health will continue to exacerbate racialised health disparities, as currently the genetic material stored in biobanks used to inform genomic precision public health is heavily skewed towards people of European ancestry [52]. The implications are that people whose ancestry is not represented in the biobanks do not benefit from advances in genomic medicine, now or in the future.

### Innovation

Global efforts are underway to diversify the genomic material stored in databases that guide genomic research and clinical practice. In Australia, the OurDNA program is a program partnering with specified multicultural communities to collect genetic information and establish more equitable genomic resources, including open-access databanks that are representative of the ancestral diversity of the Australian community [53].

### Role of implementation science

1. Report adaptations to the OurDNA program. Semistructured interviews will be conducted yearly with individuals from the various program teams (community engagement, participant recruitment, biosample collection and processing, genomic analysis, and project management). Interview data will be used to generate two process maps that outline, at each time point, the ‘actualised’ process of what has been implemented and the ‘intended’ process going forward. The maps will capture the participant journey, from the initial community engagement to participant sign-up and sample collection, finishing with genomic analysis being uploaded to the databank. Adaptations made to the process to meet the needs of each multicultural community can then be recorded and monitored using the questions from FRAME. Recording of the adaptations using FRAME will enable sharing of learnings that can be applied to other settings, saving valuable resources and time.
2. Apply an antiracism lens to recognise, revise, and reconfigure to ensure that the methods and tools used for capturing and assessing implementation outcomes related to the ‘implementability’ [47] (e.g. the acceptability) of precision public health initiatives across diverse populations are culturally inclusive [54].

### Impact (the “so what?”)

As the evidence that drives genomic medicine and clinical practice becomes more representative of diverse ancestral communities, consideration for how the data collection tools used to collect and synthesise evidence around the downstream implications for healthcare services and for families is needed, to ensure that families from all backgrounds can benefit from genomics, including precision public health initiatives.

## Conclusion and key areas for consideration

Globally, advancing racial and ethnic health equity has gained much focus in research, including implementation science. Progressing health equity necessitates approaches to identify and provide appropriate support for all groups to achieve the same access to, and benefits from, public health innovation. Through this discussion, we have identified five implementation science approaches, linked to 4 core components that are areas for further consideratation around cultural adaptation for public health innovation, as summarised in Table 2.

**Table 2:** Core elements of implementation science available for cultural adaptation and areas for research direction

Core element of implementation science	Implementation science approach to proactively facilitate cultural adaptation	Area for research direction
Development/selection/adaptations of evidence-based interventions and theories, models, and frameworks	Structure the cultural adaptation process using a formal and theory informed process	Identify an approach to (a) help select which of the existing tools developed to guide cultural adaptation to use, and (b) determine how the tools can be optimised to promote widespread use within the implementation of the public health innovation process
Stakeholder engagement	Incorporate inclusive and participatory approaches to cultural adaptation	Identify what would encourage stakeholders to participate in the adaptation process and decision-making based on what they consider valuable
Implementation strategies	Conceptualise cultural adaptation as an implementation strategy	Identify how cultural adaptation has been used as an implementation strategy to support the implementation of evidence-based public health interventions and assess the impact across health and implementation outcomes
Evaluation approaches	Evidential justice—investigate cultural adaptation of implementation science data collection tools	Identify whether current implementation science community-facing data collection methods and tools are fit for purpose and when and how they could be culturally adapted to improve cultural inclusivity

Improving the practice of adaptation as an inclusive and participatory process may support working towards implementing public health innovation that promotes health equity and provide all people with what they need to achieve better health outcomes. Implementation science, by facilitating opportunities to systematically plan for and make cultural adaptations to programs whilst measuring impact, is ideally placed to be effective at enhancing approaches to address or safeguard against unintended consequences. However, it is critical to examine aspects of how the field operates, including the practice of adaptation. Equitable implementation needs be prioritised [14]; if it is not, the cost is that implementation science risks contributing to the unintended consequences it seeks to identify and address.

## Declarations

For this article no studies with human participants or animals were performed by any of the authors. All studies mentioned were in accordance with the ethical standards indicated in each case.
